# Revealing dichotomous prior biases in social anxiety through a social prism model

**DOI:** 10.1371/journal.pcbi.1014509

**Published:** 2026-07-27

**Authors:** Yuxi Wang, Qianqian Ju, Renhe Jia, Minghao Yuan, Yujia Peng

**Affiliations:** 1 School of Psychological and Cognitive Sciences and Beijing Key Laboratory of Brain-Computer Interface and Mental Health Modulation, Peking University, Beijing, P.R.China; 2 State Key Laboratory of General Artificial Intelligence, Beijing Institute for General Artificial Intelligence, Beijing, P.R.China; 3 School of Psychology, Shanghai University of Sport, Shanghai, P.R.China; 4 Key Laboratory of Machine Perception (Ministry of Education), Peking University, Beijing, P.R.China; 5 Institute for Artificial Intelligence, Peking University, Beijing, P.R.China; Soochow University, CHINA

## Abstract

Social situations can be overwhelming for some people, triggering avoidance and social anxiety (SA). However, it remains unknown why identical situations lead to different interpretations. Here, we developed a Social Prism Model to systematically address mechanisms underlying atypical social cognition in SA within a Bayesian cognitive framework. Through eight social valence judgment experiments (N = 541), we demonstrated that social anxiety may be shaped not by how sensory evidence is processed, but by robust dichotomous prior biases depending on social cues. The dichotomous prior biases indicated two parallel mental shortcuts that predispose individuals toward social fear, where negative prior biases were associated with an intolerance to uncertainty and a fear of social evaluations, and over-positive prior biases were associated with negative social learning. Our Bayesian simulations further demonstrated how variations in prior expectations parameters can give rise to biased social judgments, providing mechanistic support for the proposed framework. Collectively, these findings showed that the negative interpretation of the social situations can be understood within the Bayesian framework, highlighting a key role of prior expectation in shaping human social cognition.

## Author summary

Social situations can be overwhelming for some individuals, yet it remains unclear why the same scene triggers very different responses across people. Through a series of empirical human studies, we systematically decomposed social scenes into social cues of faces, bodies, animacy, emotion, interaction, and ambiguity. We showed that biased social cognition in social anxiety may arise from conflicting expectations about the social world, rather than from difficulty processing social cues. People with high social anxiety traits hold negative prior biases associated with intolerance to uncertainty and a fear of social evaluations, and at the same time, overly positive prior biases that reinforce mal-adaptive social learning. Together, our work offers a theory-driven framework for understanding heterogeneity in biased social cognition among social anxiety individuals, with implications for more targeted mental health interventions.

## Introduction

**Fig 1:**
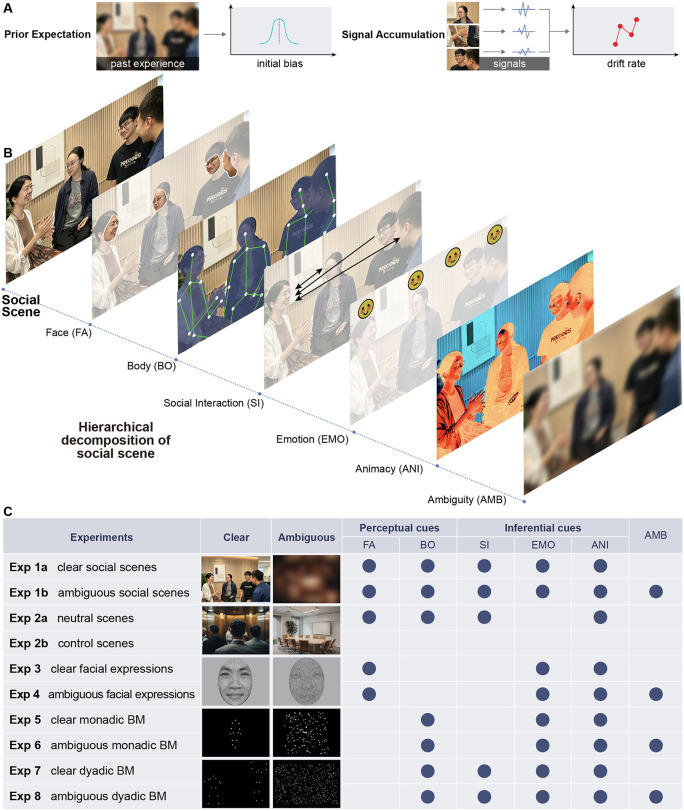
The decomposition of social scenes via a Social Prism framework. **(A)** An illustration of processes of prior expectation and signal accumulation underlying social cognition of social scenarios. **(B)** The Social Prism systematically deconstructs the complex social scenarios into hierarchical components of social cues and ambiguity. Social cues were further decomposed into perceptual cues (face, body) and inferential cues (social interaction, emotion, animacy). **(C)** Stimuli used in all the experiments and the mapping of experimental stimuli onto social cues decomposed by the Social Prism framework, with circles indicating the presence of specific social cues in each experiment. Facial and bodily cues were defined by the presence of a face or a body, respectively. Animacy was indicated by the presence of something being a living thing; social interaction by scenes involving engagement between two or more agents; and emotion by the presence of affective valence. Ambiguity was introduced through controlled visual noise or image blurring. BM = biological motion, Exp = Experiment.

Social situations can be overwhelming for some people, triggering avoidance and social anxiety (SA). However, it remains unknown why identical situations lead to different interpretations. Atypical social cognition in SA often manifests as misinterpreting social cues [1,2]. For instance, when giving a presentation, a casual frown from the audience can be perceived by some individuals as dissatisfaction with a failed presentation. In contrast, others may consider the signal random and remain unaffected. Despite the existence of numerous studies on atypical social cognition in SA, the findings have been inconsistent. Some studies have reported negative interpretation biases in SA [3,4], while others found no such bias [5] or even a positive bias [6]. These inconsistencies point to the need for a unified computational framework to systematically characterize the cognitive mechanisms underlying atypical social cognition in SA. To address this issue, social cognition can be conceptualized as involving reciprocal interactions between prior expectations and signal accumulation of social cues, which together shape our social reactions and interpretations [7,8] (Fig 1A). Prior expectations guide the interpretation of information by integrating past experiences with current cues, while the signal accumulation process incorporates key social information from actual scenarios [8]. Therefore, biases in social cognition may stem from distortions in either or both prior expectations and signal accumulation. For example, a negatively biased prior expectation can skew social interpretation [9], and over-accumulation of negative signals can similarly lead to negative interpretations [10]. Here, we propose the importance of disentangling biased prior expectations from mal-adaptive signal accumulation, as well as the specific contributions of social cues. Clarifying these mechanisms is critical for advancing both theoretical models and clinical interventions.

**Fig 2:**
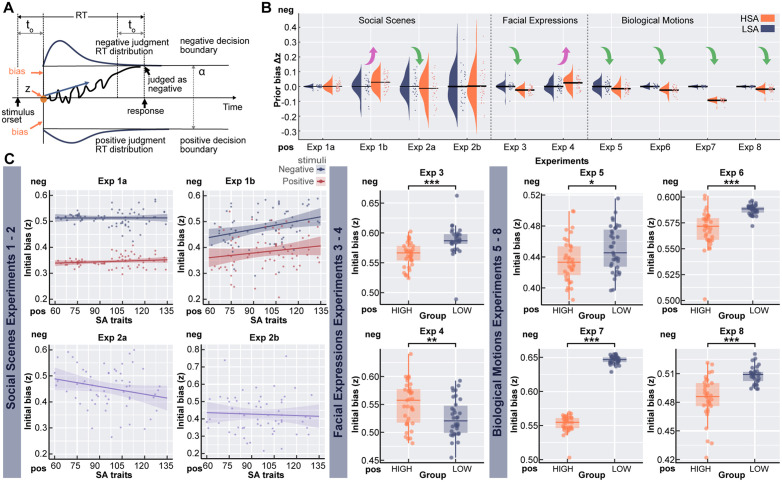
HDDM model parameters and associations with SA traits. **(A)** An illustration of the HDDM model and parameters. HDDM model depicts human decision-making as an evidence accumulation process that eventually reaches one of two boundaries, yielding four key parameters of initial bias (z), drift rate (v), threshold boundary (a), and non-decision time ($t_{\text{0}}$). **(B)** Prior biases (Δz) across eight experiments, reflecting individual deviations in prior expectations relative to the reference (Δz = 0, dashed line, the mean z-values in the LSA groups). Dots represent individual participants. Colored violin plots display the distribution of prior biases for HSA (orange) and LSA (blue) groups. Solid horizontal black lines indicate the group means of prior bias for HSA and LSA, respectively. The LSA groups were consistently centered on the no-bias reference (y = 0), while HSA groups demonstrated prior biases in several conditions. Pink arrows indicate experiments where HSA participants showed negative prior biases, whereas green arrows denote experiments where HSA participants exhibited positive prior biases. **(C)** Associations between SA traits and initial biases and drift rates across eight experiments. Solid lines indicate best-fitting linear regressions; shaded areas represent 95% confidence intervals. Box-plots display the median (central line) and interquartile range (box). Scatters denote individual participant data points. Statistical significance: *p < .05, **p < .01, ***p < .001 (Bonferroni-corrected). RT = Reaction time; neg = negative, pos = positive; BM = biological motion; HSA = high social anxiety, LSA = low social anxiety.

Computational modeling provides a principled solution to disentangle prior expectation from signal accumulation and to quantify their contributions. In the current study, we adopted the Hierarchical Drift-Diffusion Model (HDDM, Fig 2A) [11,12] to model social cognition with four key model parameters: the initial bias (z), the drift rate (v), the threshold boundary (a), and the non-decision time ($t_{\text{0}}$) [13]. The initial bias reflects a starting tendency in information processing, with a higher z indicating a predisposition to the upper-bound choice [14]. The drift rate reflects the efficiency of signal accumulation, with a greater v indicating faster signal accumulation and higher sensitivity [15]. Here, prior expectations and signal accumulation were mapped onto the initial bias and drift rate, respectively. The remaining two HDDM parameters, a and $t_{\text{0}}$, indicate the style of decision-making (impulsive or deliberate) and the time that was irrelevant to the decision-making process, respectively [16].

Furthermore, daily social interactions involve complex and heterogeneous social cues, such as multi-agent interactions, body postures, faces, and emotional signals, that may influence the social cognition of SA in different ways [17]. However, previous studies have primarily focused on isolated social cues such as facial expressions [5,18], which may oversimplify the representation of social information, especially when SA was found to associate with an avoidance of direct gaze at faces [19]. Thus, to understand atypical social cognition and the origin of social fear, it is necessary to decompose the contributions of different social cues. To systematically decompose complex social scenes into social cues while preserving the overall composite of social context, we propose a Social Prism framework in analogy to how a physical prism disperses white light into its component wavelengths (Fig 1B). The framework decomposes social scenes into perceptual cues and inferential cues, where perceptual cues consist of face (FA) and body (BO) to be directly observed, and inferential cues consist of animacy (ANI) [20], social interaction (SI), and emotion (EMO) to be inferred from perceptual cues. Furthermore, ambiguity (AMB) is incorporated into the framework to capture situations where social information is uncertain or unclear. Under clear conditions, both prior expectations and sensory accumulation contribute to social cognition. Under ambiguous conditions, however, visual signals become less reliable, and social judgements depend more heavily on prior expectations, amplifying the influence of existing biases in prior expectations on social cognition.

The current study investigates whether biased social cognition originates in distorted prior expectations or altered accumulation of social evidence. We hypothesize that biased priors are a core computational mechanism underlying social-cognitive biases, and that distinct social cues within the Social Prism framework are linked to separable prior representations, each contributing uniquely to social cognition while being integrated within a unified computational framework.

## Results

Eight behavioral experiments of social valence judgements were conducted to examine the social cognition in participants with high and low SA traits. For experimental stimuli, as shown in Fig 1C, we adopted social scenes (Experiments 1–2), isolated facial expressions (Experiments 3–4), and biological motions (Experiments 5–8). In all the experiments, participants were asked to judge the valence of the presented stimuli, for a social scene as “positive” or “negative”, or for facial expressions and biological motions as “happy” or “angry”. Participants were asked to respond as quickly and accurately as possible. Responses and reaction times (RTs) were recorded for analysis. Participants were enrolled based on the Chinese Social Anxiety Questionnaire for Adults (CSAQ-A) [21,22], which measures the SA traits of individuals. For detailed participant information, see Table 1.

**Table 1:** Demographic characteristics of participants included in each experiment.

Experiments	Group	Gender		Age		Social Anxiety Trait	
		Female	Male	*M (SD)*	Min, Max	*M (SD)*	Min, Max
Exp. 1(*N* = 73)	HSA (*N* = 45)	28 (62.22%)	17 (37.78%)	21.40 (2.24)	18,27	114.00(12.00)	95,139
	LSA (*N* = 28)	19 (67.86%)	9 (32.14%)	21.10 (2.27)	18,26	76.70(7.64)	61,92
Exp. 2(*N* = 62)	HSA (*N* = 32)	20 (62.50%)	12 (37.50%)	22.40 (3.34)	18,30	110.00 (10.70)	95,130
	LSA (*N* = 30)	20 (66.67%)	10 (33.33%)	23.10 (3.60)	18,34	76.60 (12.30)	52,94
Exp. 3(*N* = 63)	HSA (*N* = 34)	22 (64.70%)	12 (35.30%)	21.85 (2.86)	18,28	122.62 (6.20)	115,139
	LSA (*N* = 29)	20 (68.96%)	9 (31.04%)	21.55 (3.25)	18,32	62.96 (9.07)	41,73
Exp. 4(*N* = 69)	HSA (*N* = 35)	27 (77.14%)	8 (22.86%)	21.20 (2.52)	18,29	123.20 (5.46)	110,137
	LSA (*N* = 34)	23 (67.65%)	11 (32.35%)	22.60 (3.70)	18,34	67.94 (8.50)	47,80
Exp. 5(*N* = 70)	HSA (*N* = 35)	23 (65.71%)	12 (34.29%)	21.91 (2.84)	18,28	122.48 (6.18)	115,139
	LSA (*N* = 35)	23 (65.71%)	12 (34.29%)	21.65 (3.12)	18,32	65.60 (10.13)	41,80
Exp. 6(*N* = 64)	HSA (*N* = 33)	19 (57.58%)	14 (42.42%)	21.91 (2.92)	18,30	122.54 (7.34)	110,144
	LSA (*N* = 31)	17 (54.84%)	14 (45.16%)	21.51 (2.33)	18,26	71.00 (8.37)	51,81
Exp. 7(*N* = 70)	HSA (*N* = 35)	27 (77.14%)	8 (22.86%)	21.20 (2.52)	18,29	123.20 (5.46)	110,137
	LSA (*N* = 35)	24 (68.57%)	11 (31.43%)	22.60 (3.70)	18,34	67.94 (8.50)	47,80
Exp. 8(*N* = 70)	HSA (*N* = 35)	20 (57.14%)	15 (42.86%)	21.91 (2.92)	18,30	122.54 (7.34)	110,144
	LSA (*N* = 35)	20 (57.14%)	15 (42.86%)	21.51 (2.33)	18,26	71.00 (8.37)	51,81

*Note.* While most experiments recruited independent samples, some participants contributed to more than one experiment conducted within the same session (Exp. 3 & 5, Exp. 4 & 7, Exp. 6 & 8). Participant counts reflect final samples after exclusion. Across all experiments, trials with log-transformed response times exceeding ±3 SD from the participant mean were removed. In Experiments 5–8, participants with overall accuracy <80% were excluded, and trials with response times <300 ms or exceeding stimulus duration were removed. HSA = high social anxiety; LSA = low social anxiety; M = Mean; SD = Standard Deviation.

### Individual differences in behavioral judgements of social valence

On the behavioral level (i.e., responses and RTs; S2 Fig), results did not show a consistent pattern of group differences across tasks (S2 Fig). For responses, we found a significant positive association between SA traits and the ratio of negative choices only in the negative ambiguous social scenes (Exp. 1b, β= 0.238, $R^{\text{2}}$ = 0.057, *p* = .044), but not for any other experiments (*p*s > 0.05). For RTs, in the clear monadic biological motion experiment (Exp. 5), a repeated-measures analysis of variance (ANOVA) revealed a significant interaction between group and stimulus valence, *F*(1,68) = 4.651, *p* = .035, $\eta_{p}^{\text{2}}$= 0.064. Post hoc comparisons showed that both the high social anxiety (HSA) group and the low social anxiety (LSA) group recognized happy emotions more slowly than angry emotions, with this effect being more pronounced in the HSA group. No other significant group × stimulus valence interactions were observed in the remaining experiments.

### HDDM parameters reveal initial biases in SA

Among the HDDM parameters, results showed robust associations between SA traits and initial biases (z) across experiments (Fig 2C) except for clear social scenes (Exp. 1a) and control scenes (Exp. 2b). Specifically, SA traits were positively correlated with z in negative (β = 0.233, *p* = .005) and positive ambiguous social scenes (Exp. 1b, β = 0.205, *p* = .013), and negatively correlated with z in neutral scenes (Exp. 2a, β = -0.258, *p* = .043). For facial expressions, two-sample *t*-tests revealed that HSA exhibited a significantly more positive z in clear facial expressions than LSA (Exp. 3, *t*(61) = 3.916, *p* < .001, *d* = 1.012), but significantly more negative z in ambiguous facial expressions (Exp. 4, *t*(67) = 2.813, *p* = .006, *d* = 0.676). In biological motion tasks, HSA consistently demonstrated more positive z than LSA (Exp. 5,*t*(68) = 2.600, *p* = .040, *d* = 0.500; Exp. 6, *t*(62) = -5.610, *p* < .001, *d* = -1.370; Exp. 7, *t*(68) = 40.826, *p* < .001, *d* = 9.760; Exp. 8, *t*(68) = -5.900, *p* < .001, *d* = -1.410). These results suggest that initial biases may be key contributors to atypical social cognition in individuals with high SA *t*raits. However, instead of homogeneous contributions, initial biases contributed differently *t*o the biased social cognition depending on the combination of social cues. Besides z, we did not find robust group differences for other HDDM parameters (v, a, $t_{\text{0}}$) across experiments (S5 Material in S1 Text, S3, S4 Figs).

### The Social Prism Model revealed dichotomous prior biases in SA

**Fig 3:**
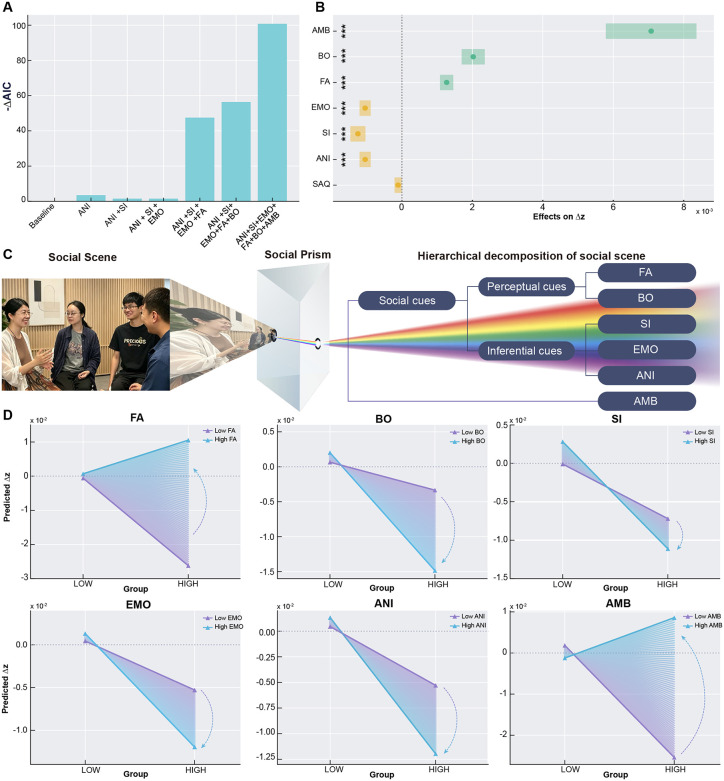
The Social Prism Model and predictive evaluation of social cognition in SA. **(A)** Linear regression model comparison. *–*Δ*AIC* for incremental addition of social cues in the Social Prism as predictors, including ANI, SI, EMO, FA, BO, and AMB. The full model, referred to as the Social Prism Model, yielded the largest –ΔAIC, indicating the best overall model fit. **(B)** Estimated effects of each social cue on Δz in the Social Prism Model. Dots indicate mean effect estimates; error bars represent 95% confidence intervals; ***p < .001. **(C)** The Social Prism systematically deconstructs the complex social scenarios into hierarchical components of social cues and ambiguity. Social cues were further decomposed into perceptual cues (face, body) and inferential cues (social interaction, emotion, animacy). **(D)** Interaction plots illustrating the estimated Δz values across levels of SA (LSA vs. HSA) and each social cue. FA = face, AMB = ambiguity, ANI = animacy, SI = social interaction, EMO = emotion, BO = body.

We quantified prior bias (∆z) as each participant’s deviation in *z* from the mean of the LSA group within the same experiment (Fig 2B; Eq. 2). We then applied a linear regression approach, incrementally adding social cue predictors to assess whether dimensions of the Social Prism explained individual differences in prior bias. The full model, which incorporated all six social cues and is hereafter referred to as the Social Prism Model (Fig 3C, Eq. 3), provided the best fit to the data (highest *− ∆AIC*; *R²* = 0.139; Fig 3A).

Regression and simple slope analyses revealed that all social cues of the Social Prism were significantly associated with prior biases in SA (Fig 3B). However, the directions of associations were not homogeneous (Fig 3D). Specifically, high SA trait was associated with **over-negative** prior biases in response to FA (*β* = 1.27 × 10^−3^, *p* < .001) and AMB (*β* = 7.08 × 10^−3^, *p* < .001), but **over-positive** prior biases in response to ANI (*β* = –1.04 × 10^−3^, *p* < .001), SI (*β* = –1.25 × 10^−3^, *p* < .001), EMO (*β* = –1.04 × 10^−3^, *p* < .001), and BO (*β* = 2.02 × 10^−3^, *p* < .001). Together, these findings demonstrate dichotomous prior biases that depend on social cues among individuals with high SA traits, highlighting the cue-specific nature of prior biases in SA.

**Fig 4:**
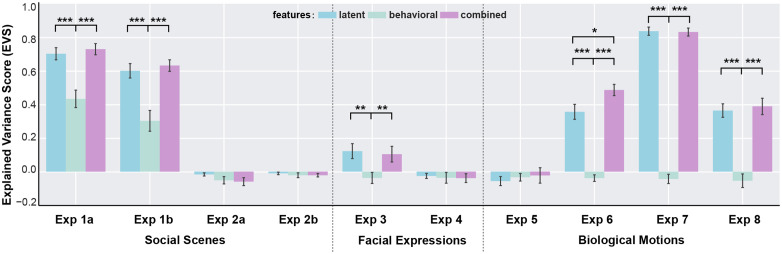
Machine learning results. Model performance comparison across feature sets using explained variance scores (EVS). Models were trained using behavioral features (green), latent features from HDDM (blue), and combined features (purple). The error bar indicates the standard error of EVS. Statistical significance: *p < .05, **p < .01, ***p < .001.

We further evaluated the performance of predicting SA traits through machine learning (ML, Fig 4). The ML model took three feature sets: behavioral features (e.g., RTs), latent features derived from HDDM parameters (e.g., z and v), and their combined features, as training inputs to predict SA traits. Results showed that latent features consistently outperformed behavioral features in predicting SA traits across experiments, except Experiments 2, 4, and 5. For instance, in Exp. 1b, latent features achieved a $M_{\text{EVS}}$ = 0.627 (${SD}_{\text{EVS}}$ = 0.199) compared to $M_{\text{EVS}}$ = 0.316 (${SD}_{\text{EVS}}$ = 0.288) for behavioral features (*t*(38) = 3.967, *p* < .001). Moreover, across all experiments except Exp. 6, models using latent features performed comparably to the combined feature models (Exp. 6, *t*(38) = 2.327, *p* = .025). For full results, see S7 Material in S1 Text and S1 Table.

### Bayesian simulation captures biased social cognition

**Fig 5:**
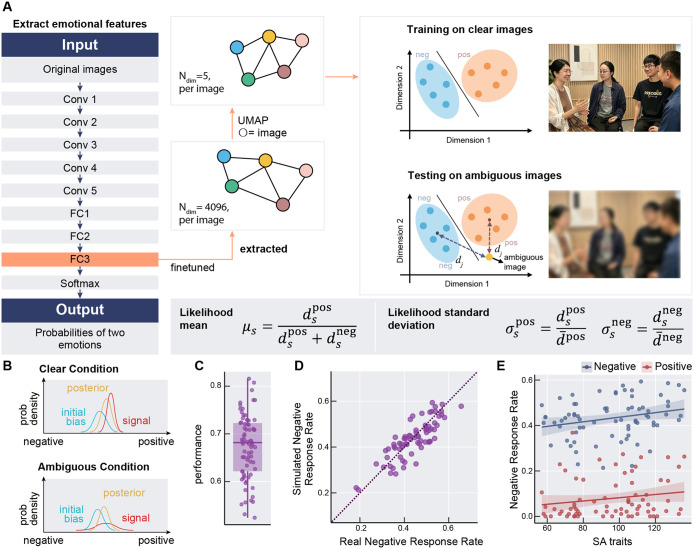
Bayesian simulation for atypical social cognition in SA under ambiguous social scene conditions. **(A)** Likelihood parameters estimation. Valence features were extracted from social scenes using a fine-tuned convolutional neural network, and projected into a low-dimensional valence space via Uniform Manifold Approximation and Projection (UMAP). A K-nearest neighbors model (KNN) trained on clear images identified the centroids of the positive and negative valence clusters, and the mean and standard deviation of the likelihood were computed based on Minkowski distances from each ambiguous image to the class centroids. **(B)** A one-step Bayesian inference framework was used to simulate participants’ binary decisions based on prior expectations and stimulus-driven likelihoods. For clear condition, prior expectation and signal accumulation both contributed to the social cognition. Under ambiguous condition, the visual signal is no longer reliable, and it heightens reliance on prior expectations during social cognition. For participant *i* and stimulus *s*, the prior was derived from the individual-level HDDM-estimated initial bias (z), while the likelihood was determined by the valence features of the stimulus. Simulated decisions were sampled from the resulting posterior distribution, and binarized based on a threshold (see Methods), where 0 indicates a positive judgement and 1 indicates a negative judgement. **(C)** Accuracy of simulated valence judgements compared to empirical valence judgements across participants. Each dot represents one individual. **(D)** Simulated negative response rates closely matched actual negative response rates across participants, indicating high generative fidelity of the model. (**E)** Simulated behavior showed that SA traits were associated with increased negative response rates, particularly to negative ambiguous stimuli, which replicated patterns observed in actual behavioral data.

To simulate biased social cognition arising from biased prior expectations, we developed a Bayesian generative model to simulate and reproduce valence judgements in ambiguous social scenes (Exp. 1b). This approach aimed to replicate the behavioral findings that HSA individuals were associated with increased ratios of negative judgements. Specifically, we estimated likelihood parameters by projecting CNN-extracted valence features into a low-dimensional space, where a K-nearest neighbors model identified cluster centroids and computed parameters based on distances to ambiguous images (Fig 5A). These likelihoods, together with prior expectations, were incorporated into a one-step Bayesian inference model to simulate participants’ binary decisions (Fig 5B).

The Bayesian simulations successfully reproduced key behavioral patterns observed in Experiment 1b. At the individual level, the simulated trial-wise responses demonstrated high accuracy when comparing model-generated responses against empirical responses as a reference (Fig 5C, M ±SD=0.674 ±0.067), significantly above chance level (*t*(72) = 22.092, *p* < .001). Simulated and actual negative response ra*t*es were highly correlated across participants (Fig 5D, *r* = 0.840, *p* < .001), demonstrating the model’s ability to capture participants’ overall response pattern. SA traits were significantly associated with simulated negative response rates for negative stimuli (β = 0.250, *p* = .033), but not for positive stimuli (β = 0.174, *p* = .140) (Fig 5E). Together, the results provide converging evidence that biased social cognition in SA can be accounted for by a Bayesian inference framework.

## Discussion

In the present study, the Social Prism Model revealed dichotomous prior biases that may drive biased social cognition in the context of SA. SA individuals showed over-negative prior biases toward facial cues and ambiguity, but over-positive prior biases in response to animacy, emotion, social interaction, and bodily cues. This dichotomous pattern may suggest a cue-specific rather than generalized fear of social scenarios underlying atypical social cognition. In particular, the negative biases associated with facial expressions may be linked to a fear of social evaluation [2], which may predispose SA individuals to automatically expect negative outcomes in response to facial cues, even in the absence of overt threat. Similarly, the negative biases associated with ambiguity may be associated with intolerance of uncertainty [23–25]. When social scenarios are ambiguous, intolerance of uncertainty in SA may trigger negative automatic thoughts such as expecting social rejection or negative judgements [26–28], thereby leading to an over-negative prior bias.

In contrast, the presence of over-positive prior biases in response to animacy, emotion, social interaction, and bodily cues may reflect atypical social learning that could reinforce strong social fear and avoidance in SA [29,30]. When facing neutral social cues (e.g., a non-smiling face), which may be more common than positive cues in daily life, an over-positive prior may lead to a negative discrepancy between the expectation and reality, contributing to disappointment or perceived rejection. Thus, counter-intuitively, the over-positive prior biases could potentially contribute to negative reinforcement learning in social scenarios for SA individuals. However, future studies are needed to empirically examine the association between prior biases and social learning.

Together, the dichotomous prior biases may help clarify the persistence of atypical social cognition in SA under repeated social exposure [26–28]. Importantly, behavioral responses emerged from the integration of prior expectations and incoming social evidence [31,32]; however, differences across social cue conditions were explained more strongly by variations in prior expectations than by differences in evidence accumulation. At a cognitive level, this finding suggests that cue-specific expectations derived from past social experiences may exert a stronger influence on social interpretations, while still operating in concert with evidence processing during social inference. For potential clinical practice, current evidence may suggest a shift of attention from social skill training to the recalibration of cue-specific prior biases. Specifically, interventions targeting over-negative priors toward facial and ambiguous cues may help reduce mal-adaptive expectations of social evaluation and rejection [33], whereas interventions addressing over-positive priors toward animacy, emotion, social interaction, and bodily cues may help recalibrate unrealistic expectations about social outcomes [34]. Such cue-specific modifications could potentially be integrated into cognitive-behavioral therapy techniques for SA [35–38] such as cognitive restructuring and related techniques [39]. As a complement, third-wave therapies, including mindfulness-based approaches [37,38,40], may align with the proposed framework by reducing the dominance of mal-adaptive priors and increasing tolerance of uncertainty through acceptance, decentering, and cognitive defusion. However, because these approaches primarily target individuals’ relationship with their thoughts and experiences, they may not directly modify the content of cue-specific priors and may therefore be insufficient to fully recalibrate prior biases when implemented alone. Future studies are needed to determine whether and how these interventions differentially modify prior expectations, evidence processing, and their integration during social inference.

Importantly, the dichotomous prior biases identified in the current study may help reconcile inconsistent behavioral findings in previous literature on atypical social cognition in the context of SA. For instance, the null effect reported by Jusyte and Schönenberg [5] may reflect the cancellation of opposing effects driven by different social cues: an over-positive prior bias triggered by emotional information and a negative prior bias associated with faces. In contrast, the negative interpretation bias observed by Heuer et al. [3] likely emerged because the stimuli initially presented neutral facial expressions that gradually morphed into emotional ones. In the absence of strong early emotional cues to elicit positive priors, the default negative bias toward faces may dominate and shape the negative interpretation. These examples highlight the importance of disentangling the specific social cues involved in shaping prior expectations. Separately, the absence of signal accumulation effects in our study may be attributable to differences in stimulus modality, as our use of rapid visual social cues compared to the verbal stimuli used in previous work [10].

In addition, Bayesian simulations in the current study accurately reproduced participants’ response patterns, supporting a cue-structured account of predictive inference, in which distinct social inputs, such as faces, bodies, or emotional information, may be associated with separable generative models. Within the Bayesian framework, these predictive processes are driven by prior expectations, which guide the interpretation of incoming social information [41–44]. Because the present simulation was implemented only in ambiguous social scenes, which incorporated all cue dimensions represented in the Social Prism framework, it should be regarded as an initial proof-of-concept demonstration. Future studies extending similar computational modeling approaches across additional social cue domains will be important for evaluating the generalizability of this account and providing broader validation of the framework.

At a neural level, the Social Prism Model may be supported by partially distinct neural pathways involving top-down regulation of different categories of social information [45–47]. Specifically, over-negative priors toward facial and ambiguous cues may be linked to threat-related networks involving the amygdala, insula, anterior cingulate cortex, and medial prefrontal cortex [48,49]. In contrast, over-positive priors toward animacy, emotion, social interaction, and bodily cues may rely more heavily on social perception and mentalizing networks, including the superior temporal sulcus, temporoparietal junction, and medial prefrontal cortex [50,51]. Future studies combining computational modeling with neuroimaging approaches may help determine how cue-specific prior biases are represented across neural systems during social inference. Furthermore, electroencephalography (EEG) and event-related potentials (ERPs) may help characterize the temporal dynamics of social inference and identify when cue-specific prior biases emerge during information processing. Early ERP components such as P1 and N170 may be particularly relevant to the perceptual processing of social cues, whereas later components such as P3 and the late positive potential (LPP) may reflect evaluative processing and belief updating [52–55]. Identifying these temporal dynamics may help clarify how prior biases unfold across processing stages and provide additional constraints for computational models of atypical social cognition.

A few limitations remain for the current study. First, the Social Prism Model is primarily limited to static social images or decontextualized dynamic biological motion stimuli. Incorporating dynamic social scenarios with greater complexity would improve its ecological validity and better capture real-world social inference. Moreover, although the Social Prism Model explained variability in cue-specific prior biases, the origins of these biases remain unclear. Factors operating at broader developmental and contextual levels, such as developmental history [56] and fluctuations in state anxiety [57], may contribute to the formation or modulation of cue-specific priors. Future work should integrate these influences to extend the model. Second, the relatively large effects observed in the biological motion experiments should be interpreted with caution. One possible explanation is that biological motion provides dynamic social information that unfolds over time, potentially increasing the influence of cue-specific prior biases on behavioral responses. Third, the current findings are based on trait-level SA in the general population, rather than clinically diagnosed cases. Due to strong social avoidance and high comorbidity of SA with depression and other anxiety disorders [2], recruiting “pure” SA patients in clinical settings remains challenging. Future studies that validate the model in clinical samples merit attention. Moreover, the identified prior biases were inferred from group-based contrasts defined by established subclinical social anxiety thresholds. Future research should examine whether these effects generalize along continuous dimensions of individual differences. Finally, the current study does not establish the causal role of biased prior expectations in atypical social cognition. Future research could address this limitation by experimentally manipulating prior probabilities [14,58].

In summary, the Social Prism Model provided a unified computational account of social anxiety. By framing biased social processing as a trade-off between cue-specific over-negative and over-positive prior expectations, the model indicated two parallel pathways contributing to the atypical social cognition in the context of SA: fear of negative evaluation resulting from over-negative expectations, and negative social learning resulting from violations of over-positive expectations. Bayesian simulations further demonstrated how individual differences in prior bias parameters shape social cognitive behaviors, providing computational support for the proposed mechanisms. This framework provided a formal foundation for translating core components of cognitive-behavioral models [26,27,59,60] into a predictive processing account of social cognition. More broadly, this work highlighted the promise of computational psychiatry that goes beyond symptomatology [61,62] and uncovered latent cognitive mechanisms, thereby motivating theory-driven interventions.

## Methods

### Ethics statement

All experiments have been approved by the Ethics Committee of Peking University (#2022-02-10). All participants provided written informed consent before participating in the experiments. Experiments were preregistered on the Open Science Framework (OSF) before data collection. The full pre-registration is available at https://osf.io/nypaz/overview?view_only=9ce4a968b6f145b5be0b7ca10f5e010f (Experiments 1–2) and https://osf.io/kyw7a/overview?view_only=aa6ea58c555d40f2884eea012ed28646 (Experiments 3–8).

### Social anxiety questionnaire

The Chinese Social Anxiety Questionnaire for Adults (CSAQ-A) [21,22] was used to measure the individual levels of SA trait. Total scores range from 30 to 150, with higher scores indicating greater SA trait levels. The normative mean for Chinese young adults was 94.93, and the cut-off scores for the high and low SA groups were 110 and 81, respectively [22]. Group definitions are provided in S1 Material in S1 Text.

### Sample size determination

Sample size determinations were based on a combination of a priori power analyses and post hoc sensitivity analyses using G*Power (Version 3.1) [63]. For Experiments 1–2, no prospective power analysis was conducted. Instead, a post hoc sensitivity analysis was performed to estimate the minimum detectable effect size for the linear regression models. To ensure a conservative estimate, we based the analysis on Experiment 2, which had the smallest sample size (*N* = 62; Exp. 1: *N* = 73). Assuming two predictors (i.e., SA trait scores and stimulus valence), *α* = .05 and 80% power, the analysis indicated sensitivity to effects of Cohen’s *f²* ≥ 0.164, corresponding to a moderate effect size. This suggests that the study had sufficient power to detect moderate effects. For Experiments 3–8, sample size planning was informed by a priori power analysis for a 2 (group) × 2 (stimulus valence) repeated-measures ANOVA. Assuming an interaction effect of *f* = 0.19, *α* = .05, 80% power, a correlation among repeated measures of 0.5, and nonsphericity correction ε = 1, the analysis indicated that a minimum sample of *N* = 58 would be sufficient.

### Valence judgement tasks and experiment design

#### Experiment 1. Clear and ambiguous social scenes.

**Participants.** In Experiment 1, we enrolled 45 HSA (28 females; age 21.40 ± 2.24 years; CSAQ-A 114.00 ± 12.00) and 28 LSA subjects (19 females; age 21.10 ± 2.27 years; CSAQ-A 76.70 ± 7.64). The two groups were matched in age and gender (Table 1). All participants had normal or corrected-to-normal vision, without any visual impairments. All participants had no current or historical psychiatric or neurological illness. Participants were compensated with ¥40–50.

**Stimuli.** Social scene images were adopted from the Social Artificial Intelligence Picture System (SAIPS) [64], with half demonstrating positive valence and half negative valence. The valence score of the images ranged from 1 (highly negative) to 9 (highly positive). The formal experiment utilized 48 positive images (*M* = 7.42) and 48 negative images (*M* = 3.85), as shown in Fig 1C. Ambiguous images were created by applying a 2D Gaussian smoothing kernel (σ = 55) to the clear images using MATLAB’s “*imgaussfit*”.

**Procedure.** Social scenes were presented with a visual angle of 10° on a screen with a resolution of 1920 × 1080 pixels and a refresh rate of 60 Hz, with a viewing distance of 70 cm. Participants first underwent a practice phase consisting of 4 trials. Each trial began with a fixation cross displayed for 800–1200 ms, followed by the presentation of a social scene. Participants were instructed to quickly and accurately judge whether the perceived valence conveyed by the image was positive or negative by pressing the left (key “F”) or right (key “J”) button.

The task consisted of 4 blocks, each containing 48 trials (24 positive stimuli and 24 negative stimuli), for a total of 192 trials. No feedback was provided during the task. The first two blocks presented clear images, while the last two involved ambiguous social scenes. Response key assignments were counterbalanced across both blocks and participants. The order of stimulus presentation within each block was randomized.

#### Experiments 2a and 2b. Neutral scenes and control scenes.

**Participants.** In Experiment 2, we enrolled 32 HSA (20 females; age 22.40 ± 3.34 years; CSAQ-A 110.00 ± 10.70) and 30 LSA subjects (20 females; age 23.10 ± 3.60 years; CSAQ-A 76.60 ± 12.30). The two groups were matched in age and gender (Table 1).

**Stimuli and Procedure.** A total of 48 neutral social scenes and 48 control scene images were adopted from SAIPS (Fig 1C) [64] with valence scores ranging from 4 to 6 (neutral: *M* = 4.67; control: *M* = 4.91). The experimental procedure was identical to that of Experiment 1. A total of 96 trials were presented in random order in two blocks, with 48 trials in each block.

#### Experiments 3–4. Clear and ambiguous facial expressions.

**Participants**. Experiment 3 followed the recruitment process and criteria as in Experiment 1. A total of 34 HSA (22 females; age  21.85 ± 2.86 years; CSAQ-A 122.62 ± 6.20) and 29 LSA participants (20 females; age 21.55 ± 3.25 years; CSAQ-A 62.96 ± 9.07) were enrolled. For Experiment 4, ambiguous face expressions, 35 HSA (27 females; age 21.20 ± 2.52 years; CSAQ-A 123.20 ± 5.46) and 34 LSA participants (23 females; age 22.60 ± 3.70 years; CSAQ-A 67.94 ± 8.50) were enrolled. Both groups were matched on age and gender across two experiments (Table 1). Participants received an average compensation of ¥35 per experiment.

**Stimuli**. A total of 45 happy and 45 angry facial expressions with balanced gender were adopted from the NimStim Set of Facial Expressions (NimStim) [65] and the Yonsei Face Database (YFace DB) [66]. All images were standardized for luminance, color contrast, and eye alignment, then converted to grayscale. For clear faces in Experiment 3 (Fig 1C), mild Gaussian noise (*M* = 0, *SD* = 0.1) was added using MATLAB’s “*imnoise*” function to mitigate the ceiling effect. More intense noise (*M* = 0, *SD* = 2) was applied to create ambiguous faces for Experiment 4 (Fig 1C).

**Procedures.** Facial expressions were presented with a visual angle of 6.4° [67]. The facial expressions were presented against a background color calculated from the average of the facial image pixels. Participants first went through a practice phase with 10 trials. Each trial started with a 0.5-second fixation cross, followed by the display of facial stimuli. Participants were asked to judge whether the perceived emotion conveyed by the facial expression was happy or angry as quickly and accurately as possible, by pressing left or right buttons. Participants were required to reach an accuracy of 0.9 in practice to proceed to the test. Otherwise, the practice phase was repeated until the criterion was met. The testing phase contained 6 blocks, each with 30 trials, yielding a total of 180 trials, with half angry and half happy stimuli. No feedback was provided during the test. On each trial, if no response was received within the 3-second response window, the trial would be repeated at the end of the test. The sequence of stimulus presentation was randomized for each participant, and button pressing was balanced across participants.

#### Experiments 5–6. Clear and ambiguous monadic biological motions.

**Participants**. 35 HSA participants (23 females; age 21.91 ± 2.84 years; CSAQ-A 122.48 ± 6.18) and 35 LSA participants (23 females; age 21.65 ± 3.12 years; CSAQ-A 65.60 ± 10.13) were recruited for the clear monadic biological motion experiment (Exp. 5). Ambiguous monadic biological motion experiment (Exp. 6) enrolled 33 HSA (19 females; age 21.91 ± 2.92 years; CSAQ-A 122.54 ± 7.34) and 31 LSA subjects (17 females; age 21.51 ± 2.33 years; CSAQ-A 71.00 ± 8.37). Both groups were matched in age and gender (Table 1).

**Stimuli**. Monadic walking point-light stimuli were adopted from the motion capture library [68] and processed using the Biomotion Toolbox in MATLAB [69]. Action instances start with a complete gait cycle and were clipped to be 8 seconds long. The videos featured a centrally positioned walker composed of 13 dots representing major human body joints, depicting an individual walking around in a circle (Fig 1C). Each of the angry and happy emotion categories included 45 action instances. Ambiguous monadic stimuli consisted of point-light walkers with embedded noisy dots (Fig 1C). The movement speed of the noisy dots followed the velocity distribution of human joint dots. Each noisy dot had a lifetime of 2–10 frames and moved either left or right. Their size matched that of the human joint dots.

**Procedures.** Point-light walkers were presented with a visual angle of 9.5°. Each biological motion stimuli were presented for 8 seconds. The other procedure and experiment settings were identical to the previous experiments.

#### Experiments 7–8. Clear and ambiguous dyadic biological motions.

**Participants**. In the clear dyadic biological motion experiment (Exp. 7), we enrolled 35 HSA subjects (27 females; age 21.20 ± 2.52 years; CSAQ-A 123.20 ± 5.46) and 35 LSA subjects (24 females; age 22.60 ± 3.70 years; CSAQ-A 67.94 ± 8.50). In the ambiguous dyadic biological motion experiment, we enrolled 35 HSA (20 females; age 21.91 ± 2.92 years; CSAQ-A 122.54 ± 7.34) and 35 LSA subjects (20 females; age 21.51 ± 2.33 years; CSAQ-A 71.00 ± 8.37). Groups were matched in age and gender (Table 1).

**Stimuli and procedure**. A total of 90 dyadic social interactive point-light stimuli were adopted from the Social Perception and Interaction Database [70], evenly split between angry and happy emotions (e.g., “A blamed B angrily, while B showed denial”). For each actor in dyadic interactions, the same 13 joint dots were extracted as in monadic walking stimuli (Experiment 6–7) through Autodesk Maya 2020 and were down-sampled to a frame rate of 60. Subsequently, actors were paired within each emotion category to compose dyadic biological motion stimuli (Fig 1C). Ambiguous dyadic interactive stimuli incorporate noisy dots matched in size and kinematic profile to joint dots, characterized by lifespans of 2–10 frames and consistent lateral displacement (Fig 1C). The procedure was identical to that of monadic biological motion experiments.

### Behavioral analysis

**Responses and RTs.** Statistical analyses were conducted using R version 4.2.1 [71]. To examine whether consistent behavioral patterns emerged across experiments in relation to SA traits, we first conducted linear regression models on the ratio of negative choices and RTs in social scene tasks (Experiments 1–2), using SA traits and stimulus valence category (negative vs. positive, or neutral vs. control) as predictors. For facial expression and biological motion tasks (Experiments 3–8), we performed repeated-measures ANOVAs on accuracy and RTs, with stimulus valence (angry vs. happy) as a within-subject factor and SA group (HSA vs. LSA) as a between-subject factor.

### Behavioral modeling using HDDM

Behavioral modeling was conducted using the *HDDM* package (version 0.8.0) [12]. We employed the stimulus-coding variant of the HDDM, in which the upper and lower decision boundaries correspond to the two response categories (Fig 2A). Participants’ responses and RTs were modeled using the Wiener First Passage Time (WFPT) distribution, the analytical solution of the HDDM with default diffusion noise.

The model included four free parameters (Fig 2A): initial bias (z), drift rate (v), threshold boundary (a), and non-decision time ($t_{\text{0}}$). z indicates the relative starting point between two decision boundaries, ranging from 0 to 1, where 0 corresponds to the lower decision boundary (positive responses) and 1 to the upper decision boundary (negative responses). A value of z=0.5 indicates no initial bias, values exceeding 0.5 indicate initial biases toward the upper decision boundary, requiring less evidence to reach the boundary and make negative judgements. The drift rate v represents the speed and direction of signal accumulation, with a larger absolute value indicating faster and more efficient processing. The sign of v denotes the direction of accumulation, where negative values reflect evidence favoring the lower decision boundary, and positive values favor the upper decision boundary. The threshold boundary a represents the total amount of evidence required to make a decision, and greater values indicate more conservative strategies. The non-decision time $t_{0}$ captures sensory encoding and motor execution latencies.

Let $t=RT-t_{\text{0}}$ denote the decision time. The WFPT density for responses terminating at the boundary is [72]:


$$\begin{array}{c} \begin{array}{c} f\left(t|\;v,a,z\right)=\frac{\pi }{a^{\text{2}}}\text{exp}\left(-vaz-\;\frac{v^{\text{2}}t}{\text{2}}\right)\;\sum_{k=\text{1}}^{\infty }k\text{exp}\left(-\;\frac{k^{\text{2}}\pi^{\text{2}}t}{\text{2}a^{\text{2}}}\right)\text{sin}(k\pi z),\;t>\text{0} \end{array} \end{array}$$ (1)

The specific HDDM model structure (i.e., parameterization of z, v, and a) was adapted to each experiment’s stimulus type, while retaining the same core HDDM framework to ensure comparability across experiments (for details see S2 Material in S1 Text). All models demonstrated satisfactory convergence ($\hat{R}$ ≤ 1.05) [73], and the estimated HDDM parameters were analyzed using the same statistical procedures as those applied to behavioral data.

### Modeling prior bias with the social prism

To investigate how distinct social cues in the Social Prism shape prior biases in SA, we conducted a series of nested linear regression analyses using the “*statsmodels*” package (version 0.14.1) [74]. The dependent variable was the prior bias, denoted as Δz, computed as the difference between a participant’s initial bias and the LSA group mean in the same experiment (Fig 2B), as follows:


$$\begin{array}{c} \begin{array}{c} \Delta {\text{z}}_{\text{i},\text{e}}={\text{z}}_{\text{i},\text{e}}-\frac{\text{1}}{\left|{\text{L}}_{\text{e}}\right|}\sum_{\text{x}\in {\text{L}}_{\text{e}}}{\text{z}}_{\text{x},\text{e}}\; \end{array} \end{array}$$ (2)

where i indexes the individual participant, e denotes the experiment, and $L_{e}$ refers to the set of LSA participants in experiment e.

We estimated six nested linear models of increasing complexity (Models 1– 6; see S3 Material in S1 Text). Model 6, referred to as the Social Prism Model, incorporated all hypothesized social cues and their interactions with SA traits, as formulated in Eq 3:


$$\begin{array}{c} \begin{array}{c} \Delta z=\beta_{\text{0}}+SA\cdot \;\left(\beta^{\text{T}}𝐗\;+\;AMB\cdot \;\gamma^{\text{T}}{𝐗}_{AMB}\;\right)+\;\varepsilon \; \end{array} \end{array}$$ (3)

where:


$$\begin{array}{c} \beta =\;{\left[\beta_{\text{1}},\beta_{\text{2}},\beta_{\text{3}},\beta_{\text{4}},\beta_{\text{5}},\beta_{\text{6}}\right]}^{\text{T}} \end{array}$$

$$\begin{array}{c} \gamma =\;{\left[\gamma_{\text{1}},\gamma_{\text{2}},\gamma_{\text{3}},\gamma_{\text{4}},\gamma_{\text{5}}\right]}^{\text{T}} \end{array}$$

$$\begin{array}{c} 𝐗=\;[\text{ANI},\text{ SI},\text{EMO},\text{FA},\text{BO},\text{AMB}{]}^{\text{T}} \end{array}$$

$$\begin{array}{c} {𝐗}_{\text{AMB}}=\;[\text{ANI},\text{ SI},\text{EMO},\text{FA},\text{BO}{]}^{\text{T}} \end{array}$$

In all models, social cues, including ANI, SI, EMO, FA, BO, and AMB, served as moderators of SA’s effect on prior biases. All binary-coded cues (ANI, SI, EMO, FA, BO) were categorical. AMB was treated as a continuous predictor and calculated using principles from signal detection theory (S4 Material in S1 Text, S1 Fig), reflecting the degree to which positive and negative interpretations of a social stimulus could be distinguished, with higher values indicating greater ambiguity. AMB was modeled both as a main effect and as a moderator interacting with other social cues, with the corresponding results reported in S6 Material in S1 Text. Categorical predictors were dummy-coded, and continuous predictors were used in their raw form.

Model performance was assessed by comparing the goodness-of-fit across models using the Akaike Information Criterion (AIC). Improvements in model fit were evaluated based on changes in AIC (*−ΔAIC*) relative to a baseline intercept-only model. Nested models were compared sequentially, and a greater decrease in AIC indicated better explanatory power. This approach enabled a principled evaluation of the additional value contributed by incorporating individual social cues and their higher-order interactions with SA.

### Predicting social anxiety scores with machine learning

We implemented eXtreme Gradient Boosting (XGBoost) using the “*scikit-learn*” package (version 1.7.0) [75] in Python to predict individual SA traits from behavioral and cognitive-level features. XGBoost was selected for its effectiveness in handling multicollinearity and delivering robust performance in structured behavioral datasets [76]. The output variable was the SA traits. Three batches of input predictors were selected to examine the contributions to SA trait prediction: behavioral features only, computational features estimated via HDDM only, and a combined set comprising both. Demographic variables were included in all three clusters.

For each stimulus type, separate models were trained and evaluated using repeated hold-out (Monte Carlo cross-validation). Data were randomly split into training (80%) and testing (20%) sets across 20 independent iterations to provide a robust estimate of model performance. Within each iteration, all preprocessing and model selection were restricted to the training set. Feature and target variables were standardized on the training data and applied to the test data. XGBoost hyperparameters were optimized using Bayesian optimization, with 5-fold cross-validation on the training set as the optimization objective. Optimization proceeded for up to 100 iterations or until convergence of the mean squared error [77]. The best hyperparameter configuration was then refit on the full training set and evaluated once on the held-out test set, which was not accessed during preprocessing, tuning, or model selection. Model performance was evaluated using the average Explained Variance Score (EVS) across all cross-validation splits. EVS measured the proportion of variance in the target variable explained by the model, with values ranging from negative infinity to 1.0. Higher EVS values indicated better predictive performance. EVS scores were compared between feature sets using an independent-samples t-test within each experiment.

### Social cognition stimulation with a Bayesian framework

Bayesian simulations were implemented in Python to model social cognitive processes in ambiguous social scenes (Exp. 1b), following the Bayesian inference framework. This experiment was selected because it incorporated the full set of social cues defined in the Social Prism and exhibited distinct behavioral patterns. Valence judgements were formalized as a one-step Bayesian inference process [41], in which trial-wise posterior distributions were computed by combining prior and likelihood distributions, both modeled as Gaussian (Fig 5B). A one-step framework was adopted because each ambiguous social scene was treated as an independent event, with judgments shaped by relatively stable prior expectations rather than sequential belief updating across trials.

**Prior Specification.** For each trial, the prior mean $\mu_{\text{prior}}$ was defined by the participant’s HDDM-estimated initial bias z conditioned on the stimulus valence. Specifically, each participant i had two valence-specific initial bias parameters, $z_{\text{neg},i}$ and $z_{\text{pos},i}$, corresponding to the negative and positive conditions, respectively. These parameters were interpreted as category-specific prior expectations reflecting stable tendencies developed across repeated encounters with negative and positive social stimuli. Let val ∈ {neg, pos} denote the valence of the stimulus. The valence-dependent initial bias for $z_{\text{val},i}$ was defined as:


$$\begin{array}{c} \begin{array}{c} z_{\text{val},i}=\left\{ \begin{array}{c} z_{\text{neg},i},\text{ val}=\text{neg}\\ z_{\text{pos},i},\text{ val}=\text{pos} \end{array}\;\right.\; \end{array} \end{array}$$ (4)

The trial-wise prior mean was then set to this value:


$$\begin{array}{c} \begin{array}{c} \mu_{\text{prior}}=\;z_{\text{val},i}\; \end{array} \end{array}$$ (5)

The prior distribution was assumed to follow a normal distribution with this mean and a fixed standard deviation of σ=0.04 to reflect moderate uncertainty:


$$\begin{array}{c} \begin{array}{c} \text{Prior}\sim N\left(z_{\text{val},i}\;,\sigma^{\text{2}}\;\right)\; \end{array} \end{array}$$ (6)

Likelihood Estimation. To derive likelihood estimates for ambiguous social scenes, we implemented a VGG16–UMAP–KNN pipeline. This approach was intended as a computational approximation of sensory evidence rather than a biologically realistic model of human perceptual processing. Likelihoods were derived from the valence features of ambiguous social scenes (Fig 5A), estimated via a K-Nearest Neighbors (KNN) classifier trained on clear social scenes with known valence labels. Valence features were extracted using a fine-tuned VGG16 convolutional neural network [78], which achieved an accuracy of 81.82% in valence judgements for clear social scenes. Feature representations were taken from the final fully connected layer (1 × 4096), and subsequently reduced to a lower-dimensional space (1 × 5) using Uniform Manifold Approximation and Projection (UMAP) [79]. Dimensionality reduction was performed separately for clear and ambiguous images to account for distributional differences. The KNN classifier, trained on clear-scene embeddings and corresponding valence labels, was applied to ambiguous-scene embeddings to produce probabilistic valence estimates.

For each ambiguous scene stimulus s, the likelihood mean $\mu_{s}$ was defined based on its normalized Minkowski distances to the positive and negative cluster centroids in the 5-dimensional UMAP embedding space:


$$\begin{array}{c} \begin{array}{c} \mu_{s}=\frac{d_{s}^{\text{pos}}}{d_{s}^{\text{pos}}+d_{s}^{\text{neg}}}\; \end{array} \end{array}$$ (7)

where $d_{s}^{\text{pos}}$ and $d_{s}^{\text{neg}}$ denote the Minkowski distances from ambiguous stimulus s to the centroids of the positive and negative clusters, respectively. This measurement reflected the relative position of the stimulus along the positive-negative valence dimension, and was used as the mean for both types of likelihood distributions.

To quantify classification uncertainty, valence-specific standard deviations were defined as normalized deviations from each cluster center:


$$\begin{array}{c} \begin{array}{c} \sigma_{s}^{\text{pos}}=\frac{d_{s}^{\text{pos}}}{{\overset{―}{d}}^{\text{pos}}}\; \end{array} \end{array}$$ (8)

$$\begin{array}{c} \begin{array}{c} \sigma_{s}^{\text{neg}}=\frac{d_{s}^{\text{neg}}}{{\overset{―}{d}}^{\text{neg}}}\; \end{array} \end{array}$$ (9)

where ${\overset{―}{d}}^{\text{pos}}$ and ${\overset{―}{d}}^{\text{neg}}$ denoted the mean intra-cluster distances of the positive and negative training samples, respectively. To prevent the likelihood from dominating the prior due to differences in scale, we normalized all likelihood standard deviations by a scaling factor λ, set equal to the prior standard deviation (λ = 0.04), and then divided by the average unnormalized standard deviation across all stimuli. The average unnormalized standard deviation $\overset{―}{\sigma }$ was computed as follows:


$$\begin{array}{c} \begin{array}{c} \overset{―}{\sigma }=\frac{\text{1}}{\text{2}}\;(\frac{\text{1}}{N}\;\sum_{s=\text{1}}^{N}\sigma_{s}^{\text{pos}}\;+\;\frac{\text{1}}{N}\;\sum_{s=\text{1}}^{N}\sigma_{s}^{\text{neg}}\;) \end{array} \end{array}$$ (10)

where N denoted the number of ambiguous images. The normalized standard deviations for positive samples ${\tilde{\sigma }}_{s}^{\text{pos}}$and negative samples ${\tilde{\sigma }}_{s}^{\text{neg}}\;$were calculated as:


$$\begin{array}{c} \begin{array}{c} {\tilde{\sigma }}_{s}^{\text{pos}}=\frac{{\lambda \sigma }_{s}^{\text{pos}}}{\overset{―}{\sigma }}\; \end{array} \end{array}$$ (11)

$$\begin{array}{c} \begin{array}{c} {\tilde{\sigma }}_{s}^{\text{neg}}=\frac{{\lambda \sigma }_{s}^{\text{neg}}}{\overset{―}{\sigma }}\; \end{array} \end{array}$$ (12)

The final likelihood distribution used for trial-level simulation was defined as a Gaussian with mean $\mu_{s}$, and a standard deviation determined by the valence of the presented stimulus (val ∈ {neg, pos}, as defined above). Specifically:


$$\begin{array}{c} \begin{array}{c} @l{\text{Likelihood}}_{s}\;\sim \;\left\{ \begin{array}{c} @lN{\left(\mu_{s},\;{(\tilde{\sigma }}_{s}^{\text{neg}}\;\right)}^{\text{2}}),\text{ val}=\text{neg},\;\\ N{\left(\mu_{s},\;{(\tilde{\sigma }}_{s}^{\text{pos}}\right)}^{\text{2}}),\text{ val}=\text{pos}. \end{array}\right.\; \end{array} \end{array}$$ (13)

All prior and likelihood distributions were truncated and normalized to the interval [0, 1], as both $\mu_{\text{prior}}$ and $\mu_{s}\;$reside in a bounded decision space, ensuring compatibility in probabilistic integration.

**Posterior Computation and Decision Rule.** For each trial, the posterior distribution was computed by pointwise multiplication of the normalized prior and likelihood probability density functions (Fig 5B), followed by normalization:


$$\begin{array}{c} \begin{array}{c} \text{Posterior}(x)=\;\frac{\text{Prior}(x)\cdot \text{Likelihood}(x)}{\int_{\text{0}}^{\text{1}}\text{Prior}(x)\cdot \text{Likelihood}(x)dx}\;x\;\in \;[\text{0},\text{1}]\; \end{array} \end{array}$$ (14)

A single value $x_{sample}$ was then drawn from the resulting posterior distribution:


$$\begin{array}{c} \begin{array}{c} x_{\text{sample}}\;\sim \text{ Posterior}(x)\; \end{array} \end{array}$$ (15)

Based on this sampled value, the simulated binary decision $\hat{r}$ as generated using a fixed threshold at 0.5, where 0 indicates a positive judgement and 1 indicates a negative judgement:


$$\begin{array}{c} \begin{array}{c} \;\hat{r}=\left\{ \begin{array}{cc} \text{0},\; & x_{\text{sample}}<\text{0}.\text{5}\;\\ \;\text{1},\; & x_{\text{sample}}\geq \text{0}.\text{5}\; \end{array}\;\right.\; \end{array} \end{array}$$ (16)

**Simulation Evaluation.** Given that the simulation was parameterized using HDDM-derived priors estimated from the observed behavioral data, it was intended as a test of generative adequacy rather than an independent out-of-sample prediction. Simulation performance was evaluated in three aspects. First, for each participant, we calculated the accuracy of the model-simulated binary responses compared to the participants’ observed responses, and conducted a one-sample *t*-test against 0.5 to evaluate whether the model generated random responses. Next, we assessed the correlation between the simulated and empirical negative response rates to examine the model’s ability to capture overall response patterns. Third, to determine whether the simulations reflected key behavioral signatures, we computed the simulated negative response rate for each condition and participant. The simulated negative response rates were then entered into a linear regression model with SA traits and stimulus valence (positive vs. negative) as predictors, testing whether the simulated data replicated the SA-by-valence interaction observed in the empirical data.

## Supporting information

S1 Text S1 Material. Social anxiety group classification. S2 Material. HDDMs model specification. S3 Material. Linear regression models for predicting prior biases from social cues. S4 Material. Calculation of the ambiguity index. S5 Material. Additional hierarchical drift-diffusion model results. S6 Material. Ambiguity modulates cue-specific prior biases in SA. S7 Material. Machine learning predictive performance.
(DOCX)

S1 Fig Ambiguity values for ten stimulus types across experiments. Exp = Experiment.
(TIF)

S2 FigBehavioral results and associations with SA traits.Results did not show a consistent pattern of group differences across tasks. For responses, we found a significant positive association between SA traits and the ratio of negative choices only in the negative ambiguous social scenes (β= 0.238, $R^{\text{2}}$ = 0.057, *p* = .044), but not for any other experiments (*ps* > 0.05). For RTs, in the clear monadic biological motion experiment (Exp. 5), a repeated-measures analysis of variance (ANOVA) revealed a significant interaction between group and stimulus valence, *F*_(1,68)_ = 4.651, *p* = .035, $\eta_{p}^{\text{2}}$= 0.064. Post hoc comparisons showed that both the high social anxiety (HSA) group and low social anxiety (LSA) group recognized happy emotions more slowly than angry emotions, with this effect being more pronounced in the HSA group. No other significant group × stimulus valence interactions were observed in the remaining experiments. Solid lines represent linear regressions; shaded areas indicate 95% confidence intervals. Boxplots show medians and interquartile ranges; individual points reflect participants. Statistical significance: *p < .05, **p < .01, ***p < .001 (Bonferroni-corrected). BM = biological motion; HSA = high social anxiety, LSA = low social anxiety.(TIF)

S3 FigDrift rate results and associations with SA traits.Results did not show a consistent pattern of group differences across tasks. HDDM results only revealed a faster v of negative signals in the HSA group compared to LSA group in the clear monadic biological motion experiment. Solid lines represent linear regressions; shaded areas indicate 95% confidence intervals. Boxplots show medians and interquartile ranges; individual points reflect participants. Statistical significance: *p < .05 (Bonferroni-corrected). BM = biological motion; HSA = high social anxiety, LSA = low social anxiety.(TIF)

S4 FigDecision boundary and non-decision time results and associations with SA traits.The HSA group exhibited more conservative decision-making in the neutral scenes (β = -0.376, *p* = .003) and the clear dyadic biological motions experiment (*t*_(68)_ = -5.188, *p* < .001, Cohen’s d = 1.24). No other significant associations between SA traits and HDDM parameters were observed across experiments. Solid lines represent linear regressions; shaded areas indicate 95% confidence intervals. Boxplots show medians and interquartile ranges; individual points reflect participants. Statistical significance: *p < .05, **p < .01, ***p < .001 (Bonferroni-corrected). BM = biological motion; HSA = high social anxiety, LSA = low social anxiety.(TIF)

S1 Table Machine learning predictive performance (*M ± SD*) and statistical comparisons (*t*, *p*) between feature sets in predicting social anxiety trait scores. (XLSX)
