# Supplementary material for: Revealing dichotomous prior biases in social anxiety through a social prism model
Source: PLoS Comput Biol. 2026 Jul 27;22(7):e1014509. doi: 10.1371/journal.pcbi.1014509 (PMC13423177; doi:10.1371/journal.pcbi.1014509)
Supplement: S1 Text — S2 Material. HDDMs model specification. S3 Material. Linear regression models for predicting prior biases from social cues. S4 Material. Calculation of the ambiguity index. S5 Material. Additional hierarchical drift-diffusion model results. S6 Material. Ambiguity modulates cue-specific prior biases in SA. S7 Material. Machine learning predictive performance. (DOCX) [file pcbi.1014509.s001.docx]

**Supplementary Materials**

**Revealing Dichotomous Prior Biases in Social Anxiety through a Social Prism Model**

## **Supplementary Methods**

### **S1. Social anxiety group classification**

Social anxiety (SA) traits were measured using the Chinese Social Anxiety Questionnaire for Adults (CSAQ-A) [1,2]. In Experiments 1–2, participants were recruited across the full range of SA traits to examine the relationship between SA traits and atypical social cognition, and they were classified into the high social anxiety (HSA) and low social anxiety (LSA) groups using the normative mean score of 94.93 for Chinese young adults [2]. In Experiments 3–8, group assignment followed the cut-off scores reported in Wang et al. [2]: HSA was defined as CSAQ-A ≥ 110, and LSA as CSAQ-A ≤ 81.

### **S2. HDDMs model specification**

We constructed separate Hierarchical Drift-Diffusion Models (HDDM) for different experiments to capture the specific decision dynamics associated with each stimulus type. All parameters were estimated separately for the HSA and LSA groups.

**Experiment 1: Clear and ambiguous social scenes**

Given the complexity and contextual richness of social scenes, we assumed that participants formed valence-dependent initial bias $z$ within each condition. Accordingly, for each condition $c \in\left\{ clear, ambiguous \right\}$, we estimated separate initial biases $z_{c,\mathrm{pos}}$ and $z_{c,\mathrm{neg}}$, threshold boundaries $a_{c, \mathrm{pos}}$ and $a_{c,\mathrm{neg}}$, non-decision times ${t_{0}}_{c, \mathrm{pos}}$ and ${t_{0}}_{c,\mathrm{neg}}$, together with a single drift rate magnitude $v_{c}$ shared across stimulus valence within each condition. In the stimulus-coding scheme, positive stimuli were assigned to a drift rate ${+v}_{c}$, while negative stimuli were assigned to a drift rate ${-v}_{c}$. Decision time was defined as $t=RT-t_{0,c,s}$, where $s \in\left\{ pos, neg \right\}$ indexes stimulus valence. The Wiener First Passage Time (WFPT) density for responses terminating at the decision boundary is given by Navarro & Fuss (*3*):

$$\begin{aligned} f\left( t| v,a,z \right)=\frac{\pi}{a^{2}}\exp\left( -vaz- \frac{v^{2}t}{2} \right) \sum_{k=1}^{\infty} k\exp\left( - \frac{k^{2}\pi^{2}t}{2a^{2}} \right)\sin\left( k\pi z \right), t>0 \#\left( S2.1 \right) \end{aligned}$$

For each condition $c$, the specific boundary densities were:

Positive stimuli (${+v}_{c}$): $f_{c,\mathrm{pos}}\left( t \right)= f\left( RT-t_{0,c,\mathrm{pos}}| v_{c},a_{c,\mathrm{pos}},z_{c,\mathrm{pos}} \right)$, $c \in\left\{ clear, ambiguous \right\}$

Negative stimuli ($-v_{c}$): $f_{c,\mathrm{neg}}\left( t \right)= f\left( RT-t_{0,c,\mathrm{neg}}| {-v}_{c},a_{c,\mathrm{neg}},z_{c,\mathrm{neg}} \right), c \in\left\{ clear, ambiguous \right\}$

**Experiment 2: Neutral and control scenes**

In contrast to Experiment 1, neutral and control scenes lack explicit valence cues. To account for potential differences in participants’ prior expectations arising from contextual variation between neutral and non-social control scenes, we estimated a separate $z_{c}$, $a_{c}$, $t_{0,c}$ for each condition ($c \in\left\{ neutral, control \right\}$). A single drift rate magnitude $v$ was shared across both conditions, reflecting a common signal accumulation process in the absence of valence cues. Decision time was defined as $t=RT-t_{0,c}$. For condition $c$, the specific boundary density was:

$f_{c}\left( t \right)= f\left( RT-t_{0,c}| v,a_{c},z_{c} \right)$*，*$c \in\left\{ neutral, control \right\}$

**Experiments 3–8: Facial expressions and biological motions**

For facial expressions and biological motions, we estimated separate drift rates $v_{\mathrm{angry}}$ and $v_{\mathrm{happy}}$ to capture differential signal accumulation trajectories for each stimulus valence (angry vs. happy). In the stimulus-coding scheme, happy stimuli were assigned to an initial bias $z$, and angry stimuli were assigned to an initial bias $1-z.$For each experiment, decision time was defined as $t=RT-t_{0}$, where $t_{0}$ denotes the non-decision time parameter. The specific boundary densities for each stimulus valence were:

Happy stimuli: $f_{\mathrm{happy}}\left( t \right)= f\left( RT-t_{0}| v_{\mathrm{happy}},a,z \right)$

Angry stimuli: $f_{\mathrm{angry}}\left( t \right)= f\left( RT-t_{0}| v_{\mathrm{angry}},a,1-z \right)$

### **S3. Linear regression models for predicting prior biases from social cues**

We fitted a series of six linear models to examine the contribution of each social cue and its interaction with SA traits in predicting prior biases $\Delta z$. The models were constructed in a stepwise manner, with each successive model including all predictors from the previous model plus one additional social cue. Models 1–5 introduced binary-coded social cues sequentially, including animacy (ANI), social interaction (SI), emotion (EMO), FA (face), BO (body)(Fig. 3A). Each social cue was included as an interaction term with SA traits. Model 6, the Social Prism Model, additionally included ambiguity (AMB) as both a main effect and a moderator, interacting with all other social cues. The linear regressions were specified as follows:

$$\begin{aligned} \Delta z=\beta_{0}+SA\cdot\left( \boldsymbol{\beta}^{T}\mathbf{X} \right)+ \varepsilon\#\left( S4.1 \right) \end{aligned}$$

$$\begin{aligned} \Delta z=\beta_{0}+SA\cdot\left( \boldsymbol{\beta}^{T}\mathbf{X} + AMB\cdot\boldsymbol{\gamma}^{T}\mathbf{X}_{AMB} \right)+ \varepsilon\#\left( S4.2 \right) \end{aligned}$$

Here, $\mathbf{X}$ is the vector of binary cues included in the model, and $\boldsymbol{\beta}$ is the corresponding coefficient vector. In Eq. (S4.2), $\mathbf{X}_{AMB}$ denotes the cues excluding AMB, and $\boldsymbol{\gamma}$ is the coefficient vector for the three-way interaction terms $SA\cdot AMB\cdot\mathbf{X}_{AMB}$. Bold lowercase letters denote column vectors, $\left( \cdot\right)^{T}$ is the transpose.

**Model 1 (ANI) — Eq. (S4.1)**

$$\boldsymbol{\beta=}\left[ \beta_{1} \right]^{T}$$

$\mathbf{X=}\left[ \mathrm{ANI} \right]^{T}$

**Model 2 (ANI + SI) — Eq. (S4.1)**

$$\boldsymbol{\beta=}\left[ \beta_{1},\beta_{2} \right]^{T}$$

$\mathbf{X=}\left[ ANI, SI \right]^{T}$

**Model 3 (ANI + SI + EMO) — Eq. (S4.1)**

$$\boldsymbol{\beta=}\left[ \beta_{1},\beta_{2},\beta_{3} \right]^{T}$$

$\mathbf{X=}\left[ ANI, SI,EMO \right]^{T}$

**Model 4 (ANI + SI+ EMO + FA) — Eq. (S4.1)**

$$\boldsymbol{\beta=}\left[ \beta_{1},\beta_{2},\beta_{3},\beta_{4} \right]^{T}$$

$\mathbf{X=}\left[ ANI, SI,EMO,FA \right]^{T}$

**Model 5 (ANI + SI+ EMO + FA + BO) — Eq. (S4.1)**

$$\boldsymbol{\beta=}\left[ \beta_{1},\beta_{2},\beta_{3},\beta_{4},\beta_{5} \right]^{T}$$

$\mathbf{X=}\left[ ANI, SI,EMO,FA,BO \right]^{T}$

**Model 6 Social Prism Model (ANI + SI+ EMO + FA + BO + AMB) — Eq. (S4.2)**

$$\boldsymbol{\beta=}\left[ \beta_{1},\beta_{2},\beta_{3},\beta_{4},\beta_{5},\beta_{6} \right]^{T}$$

$\boldsymbol{\gamma=}\left[ \gamma_{1},\gamma_{2},\gamma_{3},\gamma_{4},\gamma_{5} \right]^{T}$

$\mathbf{X=}\left[ ANI, SI,EMO,FA,BO,AMB \right]^{T}$

$$\mathbf{X}_{\mathrm{AMB}}\mathbf{=}\left[ ANI, SI,EMO,FA,BO \right]^{T}$$

### **S4. Calculation of the ambiguity index**

We computed the ambiguity (AMB) value of each stimulus type across the eight experiments using principles from signal detection theory [4]. Specifically, AMB was defined as the proportion of overlap between the Gaussian signal and noise distributions, normalized by the total area under the signal distribution. Both positive and negative stimuli were treated as signals. The AMB index quantifies the relative indistinguishability of valence-relevant information from noise, ranging from 0 (fully separable) to 1 (completely overlapping), with higher values indicating greater ambiguity. For neutral and control stimuli used in Experiments 2a and 2b, which lacked valence information by design, AMB was manually set to 0. AMB values for all stimuli are shown in Fig. S1.

## **Supplementary Results**

### **S5. Additional Hierarchical Drift-Diffusion Model results**

Participants’ responses and RTs data were submitted Hierarchical Drift-Diffusion Model (HDDM) to estimate decision-making parameters, including initial bias ($z$), drift rate ($v$), threshold boundary ($a$), and non-decision time ($t_{0}$). HDDM results revealed a faster $v$ of negative signals in the HSA group compared to LSA group, but only in the clear monadic biological motion experiment (Fig. S3). For $a$ and $t_{0}$ (Fig. S4), the HSA group exhibited more conservative decision-making in the neutral scenes ($\beta$ = -0.376, *p* = .003) and in the clear dyadic biological motions experiment (*t*_(68)_ = -5.188, *p* < .001, Cohen’s d = 1.24). No other significant associations between SA traits and HDDM parameters were observed across experiments.

### **S6. Ambiguity modulates cue-specific prior biases in SA**

Within the Social Prism Model, we further examined how AMB modulated the influence of distinct social cues on prior biases in individuals with SA. Significant three-way interactions were observed for Ambiguity × Emotion × SA traits (*β* = 7.08 × 10⁻³, *p* < .001), Ambiguity × Animacy × SA traits (*β* = 7.08 × 10⁻³, *p* < .001), and Ambiguity × Social Interaction × SA traits (*β* = 1.06 × 10⁻², *p* < .001). In contrast, ambiguity appeared to attenuate the SA-related prior bias associated with perceptual cues. Specifically, the interactions Ambiguity × Face × SA traits (*β* = –9.86 × 10⁻³, *p* < .001) and Ambiguity × Body × SA traits (*β* = –2.16 × 10⁻³, *p* < .001). To further probe these interactions, simple slope analyses were conducted at ±1 standard deviation of ambiguity. Results confirmed that the ambiguity-driven increases in negative prior bias were specific to emotional and animate cues, suggesting that ambiguity selectively induces a negative shift in prior biases for animate and emotional cues

### **S7. Machine learning predictive performance**

Table S1 reported the predictive performance of three feature sets: behavioral features, latent features derived from HDDM parameters, and their combined features, in predicting individual differences in SA traits across eight experiments. Performance was indexed by explained variance scores (EVS; mean ± standard deviation), along with corresponding statistical comparisons. Consistent with the main text, latent features outperformed behavioral features in most experiments, with significant improvements observed in Exp. 1a, 1b, 3, 6, 7, and 8 (*ps* < .05). Notably, combined feature sets provided additional predictive benefit over behavioral features in Experiment 1a, 1b, 3, 6, 7, and 8 but did not significantly outperform latent features in most cases, except Exp. 6. These findings highlight the added value of latent computational features over raw behavioral data in predicting SA traits and suggest that combining features yields limited improvement beyond the latent features alone.


$\beta$ $R^{2}$ $\eta_{p}^{2}$ $v$ $\beta$

## **References**

1. Caballo VE, Salazar IC, Irurtia MJ, et al. The multidimensional nature and multicultural validity of a new measure of social anxiety: The Social Anxiety Questionnaire for Adults. *Behavior Therapy*. 2012;43(2):313-328.

2. Wang Y, Zang Y, Peng Y. Validity and reliability of the Chinese Social Anxiety Questionnaire for Adults. *Chinese Mental Health Journal*. 2024;38(08):730-736.

3. Navarro DJ, Fuss IG. Fast and accurate calculations for first-passage times in Wiener diffusion models. *Journal of Mathematical Psychology*. 2009;53(4):222-230. doi:10.1016/j.jmp.2009.02.003

4. Green DM, Swets JA, others. *Signal Detection Theory and Psychophysics*. Vol 1. Wiley New York; 1966.
