# Supplementary figures and images for: Revealing dichotomous prior biases in social anxiety through a social prism model
Source: PLoS Comput Biol. 2026 Jul 27;22(7):e1014509. doi: 10.1371/journal.pcbi.1014509 (PMC13423177; doi:10.1371/journal.pcbi.1014509)

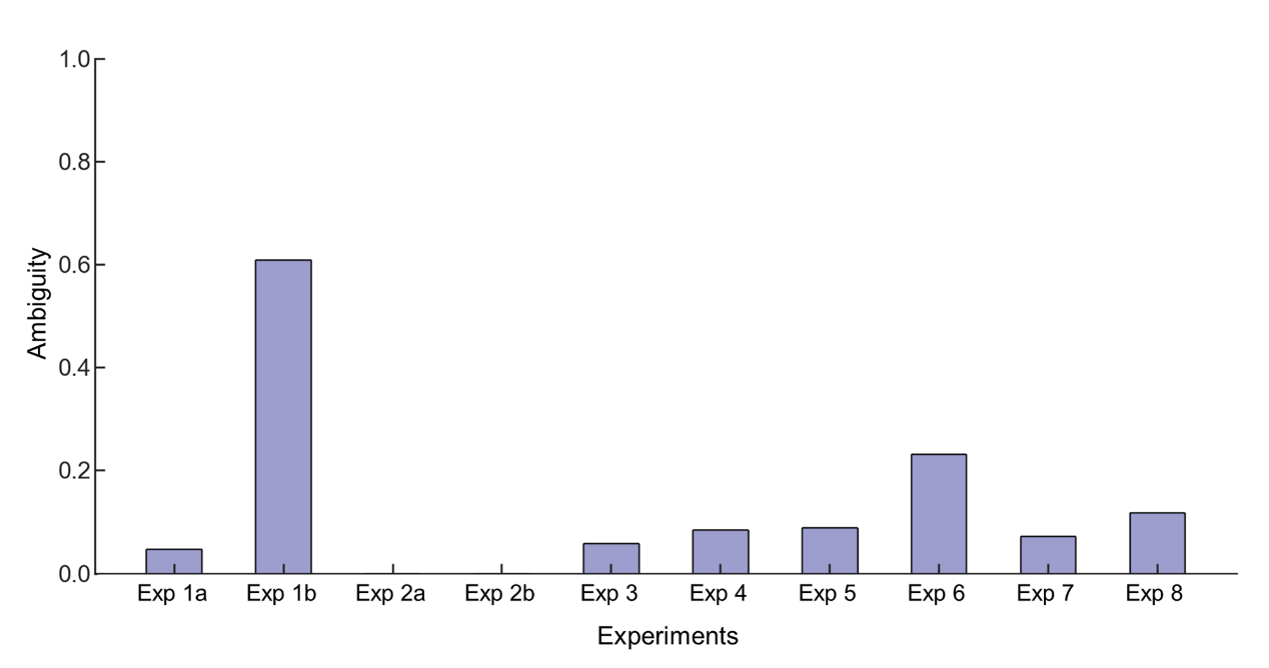

Supplement: S1 Fig — Exp = Experiment. (TIF) [file pcbi.1014509.s002.tif]

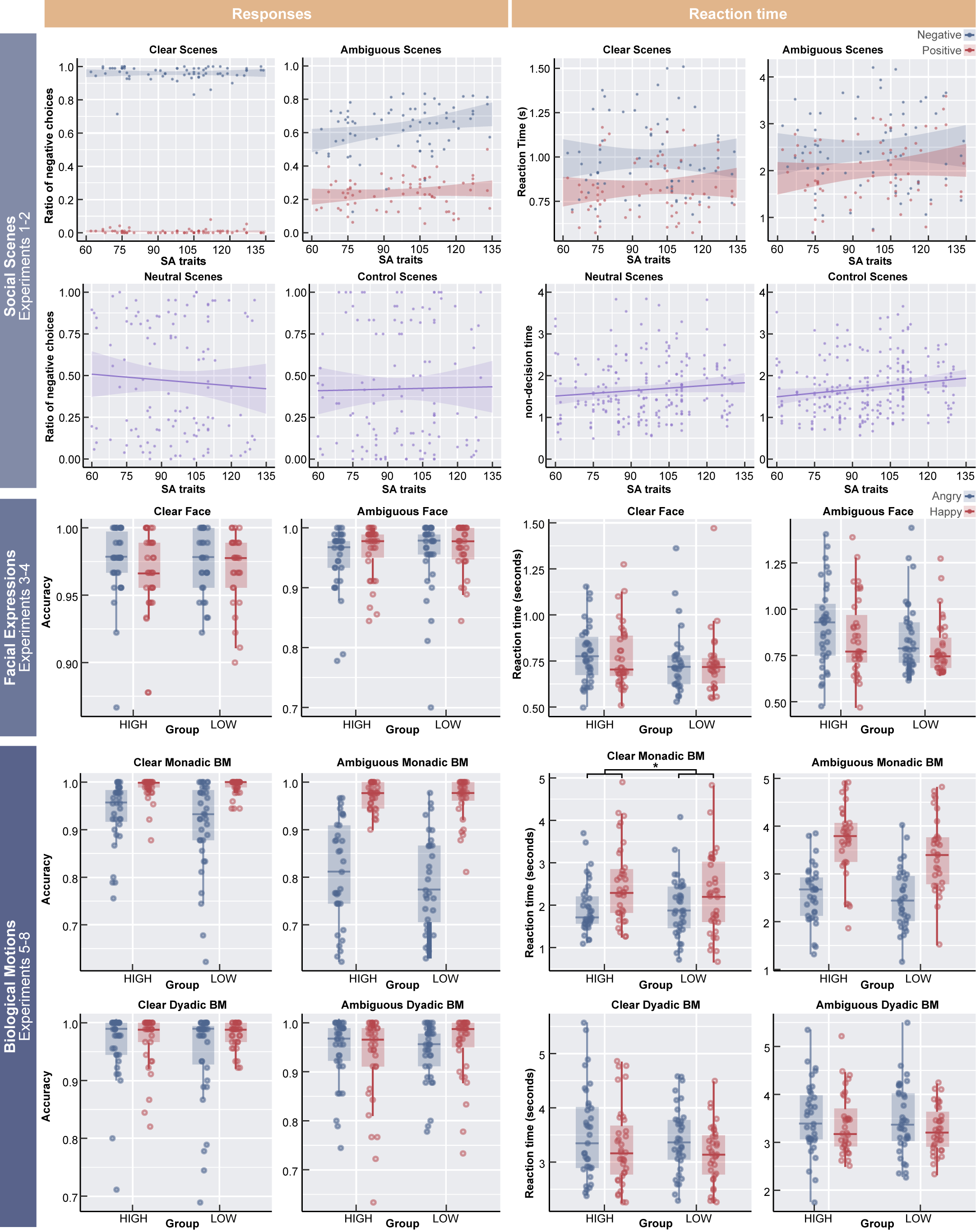

Supplement: S2 Fig — Results did not show a consistent pattern of group differences across tasks. For responses, we found a significant positive association between SA traits and the ratio of negative choices only in the negative ambiguous social scenes (β= 0.238, R2 = 0.057, p = .044), but not for any other experiments (ps > 0.05). For RTs, in the clear monadic biological motion experiment (Exp. 5), a repeated-measures analysis of variance (ANOVA) revealed a significant interaction between group and stimulus valence, F(1,68) = 4.651, p = .035, ηp2= 0.064. Post hoc comparisons showed that both the high social anxiety (HSA) group and low social anxiety (LSA) group recognized happy emotions more slowly than angry emotions, with this effect being more pronounced in the HSA group. No other significant group × stimulus valence interactions were observed in the remaining experiments. Solid lines represent linear regressions; shaded areas indicate 95% confidence intervals. Boxplots show medians and interquartile ranges; individual points reflect participants. Statistical significance: *p < .05, **p < .01, ***p < .001 (Bonferroni-corrected). BM = biological motion; HSA = high social anxiety, LSA = low social anxiety. (TIF) [file pcbi.1014509.s003.tif]

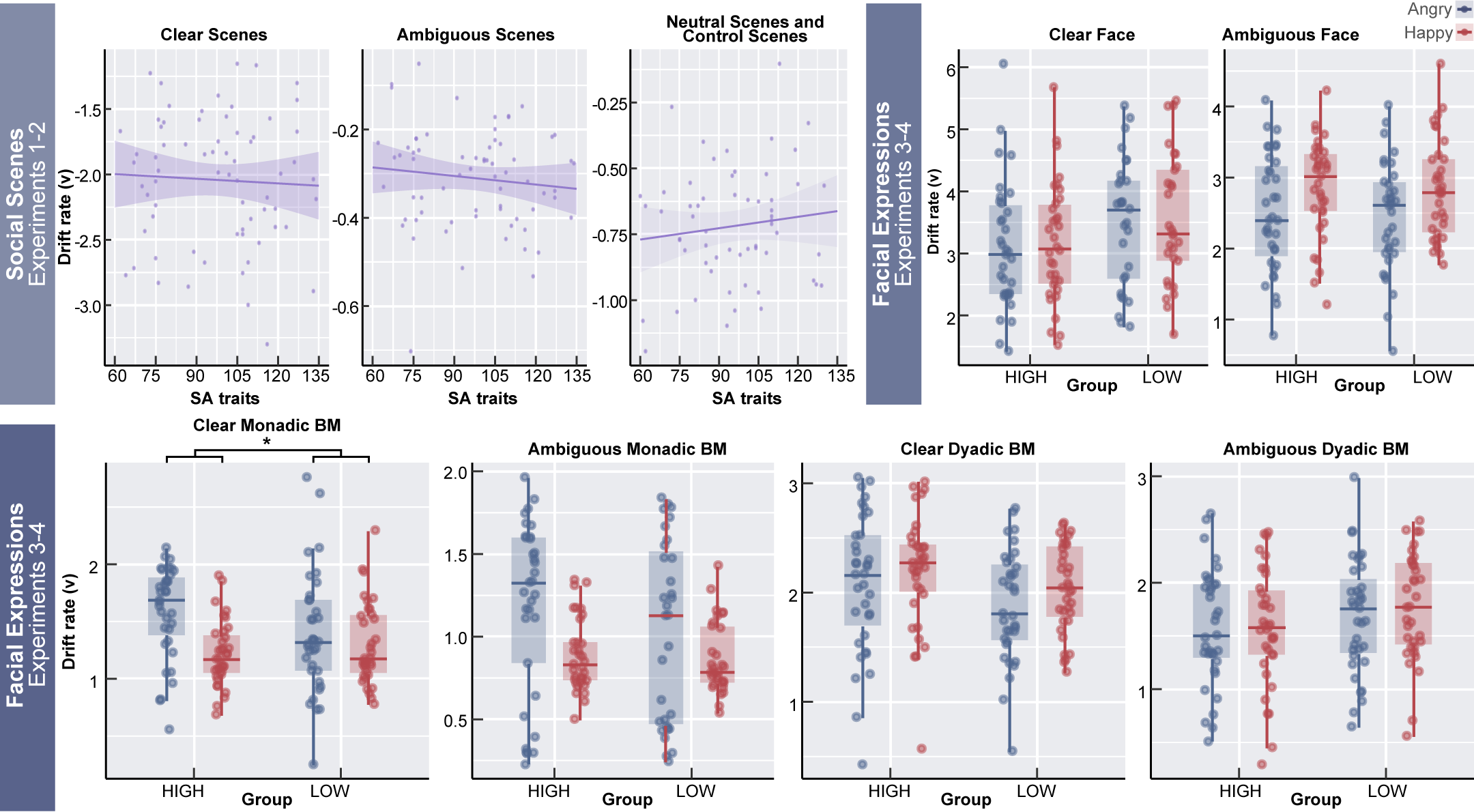

Supplement: S3 Fig — Results did not show a consistent pattern of group differences across tasks. HDDM results only revealed a faster v of negative signals in the HSA group compared to LSA group in the clear monadic biological motion experiment. Solid lines represent linear regressions; shaded areas indicate 95% confidence intervals. Boxplots show medians and interquartile ranges; individual points reflect participants. Statistical significance: *p < .05 (Bonferroni-corrected). BM = biological motion; HSA = high social anxiety, LSA = low social anxiety. (TIF) [file pcbi.1014509.s004.tif]

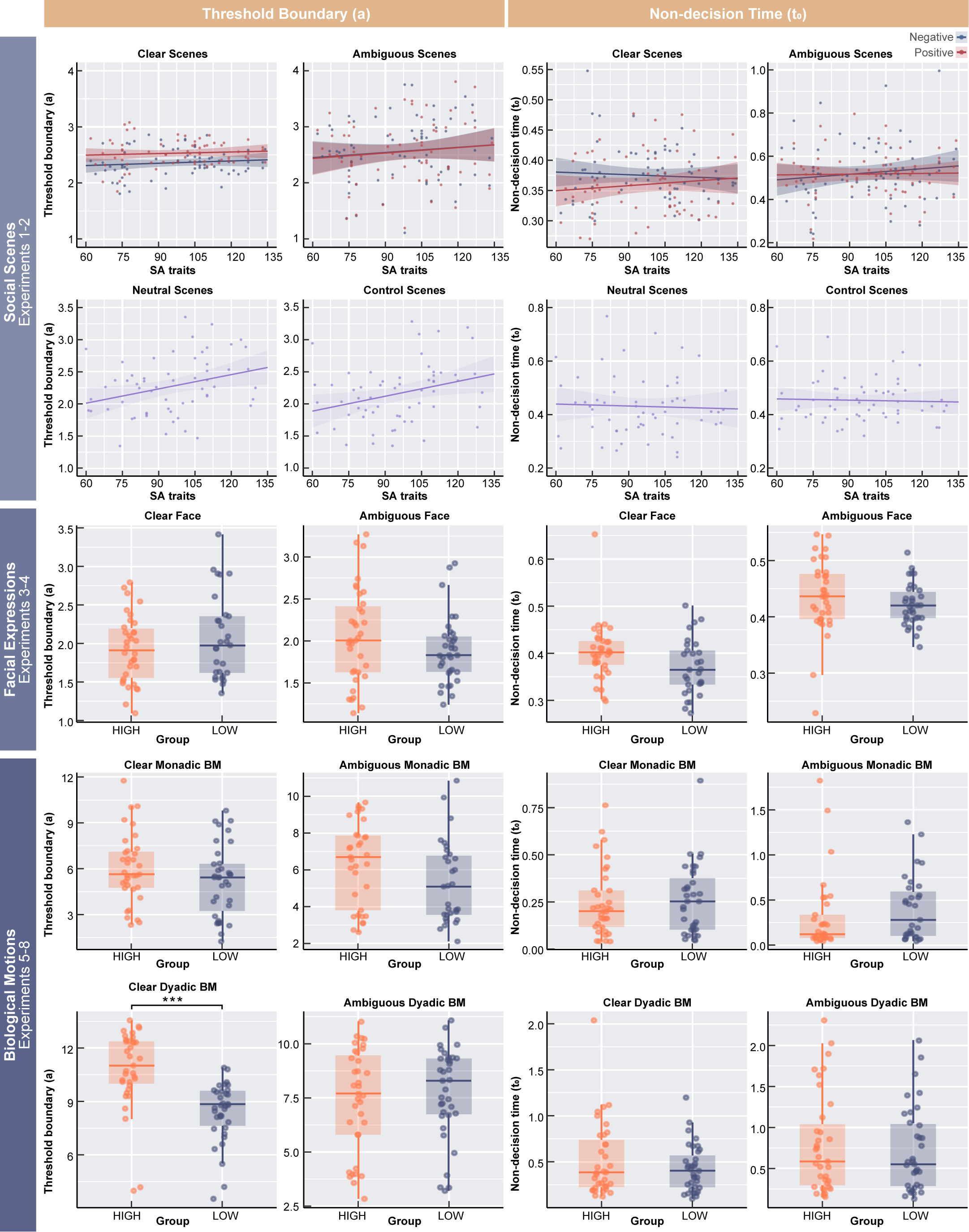

Supplement: S4 Fig — The HSA group exhibited more conservative decision-making in the neutral scenes (β = -0.376, p = .003) and the clear dyadic biological motions experiment (t(68) = -5.188, p < .001, Cohen’s d = 1.24). No other significant associations between SA traits and HDDM parameters were observed across experiments. Solid lines represent linear regressions; shaded areas indicate 95% confidence intervals. Boxplots show medians and interquartile ranges; individual points reflect participants. Statistical significance: *p < .05, **p < .01, ***p < .001 (Bonferroni-corrected). BM = biological motion; HSA = high social anxiety, LSA = low social anxiety. (TIF) [file pcbi.1014509.s005.tif]
